# *Naja atra* Cardiotoxin 3 Elicits Autophagy and Apoptosis in U937 Human Leukemia Cells through the Ca^2+^/PP2A/AMPK Axis

**DOI:** 10.3390/toxins11090527

**Published:** 2019-09-12

**Authors:** Jing-Ting Chiou, Yi-Jun Shi, Liang-Jun Wang, Chia-Hui Huang, Yuan-Chin Lee, Long-Sen Chang

**Affiliations:** 1 Institute of Biomedical Sciences, National Sun Yat-Sen University, Kaohsiung 804, Taiwan; 2 Department of Biotechnology, Kaohsiung Medical University, Kaohsiung 807, Taiwan

**Keywords:** cardiotoxin, Ca^2+^, PP2A, AMPK, autophagy, apoptosis

## Abstract

Cardiotoxins (CTXs) are suggested to exert their cytotoxicity through cell membrane damage. Other studies show that penetration of CTXs into cells elicits mitochondrial fragmentation or lysosome disruption, leading to cell death. Considering the role of AMPK-activated protein kinase (AMPK) in mitochondrial biogenesis and lysosomal biogenesis, we aimed to investigate whether the AMPK-mediated pathway modulated *Naja atra* (Taiwan cobra) CTX3 cytotoxicity in U937 human leukemia cells. Our results showed that CTX3 induced autophagy and apoptosis in U937 cells, whereas autophagic inhibitors suppressed CTX3-induced apoptosis. CTX3 treatment elicited Ca^2+^-dependent degradation of the protein phosphatase 2A (PP2A) catalytic subunit (PP2Acα) and phosphorylation of AMPKα. Overexpression of PP2Acα mitigated the CTX3-induced AMPKα phosphorylation. CTX3-induced autophagy was via AMPK-mediated suppression of the Akt/mTOR pathway. Removal of Ca^2+^ or suppression of AMPKα phosphorylation inhibited the CTX3-induced cell death. CTX3 was unable to induce autophagy and apoptosis in U937 cells expressing constitutively active Akt. Met-modified CTX3 retained its membrane-perturbing activity, however, it did not induce AMPK activation and death of U937 cells. These results conclusively indicate that CTX3 induces autophagy and apoptosis in U937 cells via the Ca^2+^/PP2A/AMPK axis, and suggest that the membrane-perturbing activity of CTX3 is not crucial for the cell death signaling pathway induction.

## 1. Introduction

Cobra cardiotoxins (CTXs) show a wide range of biological activities including hemolysis, cardiotoxicity, and disruption of muscular integrity [1,2,3]. Moreover, accumulated evidence has shown that CTXs induce apoptosis in cancer cells through the mitochondria-mediated apoptotic pathway, lysosome-mediated death pathway, or membrane-damaging effects [4,5,6,7,8]. It is widely believed that the plasma membrane is a target of CTXs, but the membrane pore formed by CTXs may have a limited lifetime on the cell surface due to membrane reorganization [9]. Therefore, some studies indicate that the cytotoxicity of CTXs is related to their capability of cell penetration [4,7,10,11]. The internalized CTXs are suggested to elicit mitochondrial fragmentation [10,11] or lysosome disruption [4,7], and thereby induce the death of the myoblast cells, primary cortical neurons, or cancer cells. Notably, Langone et al. [12] found that activated AMPK can attenuate the cytotoxicity of *Naja pallida* CTX on myoblast cells. AMPK has been demonstrated to be involved in mitochondrial biogenesis [13,14] and lysosomal biogenesis [15]. Some studies have revealed that AMPK acts as a tumor suppressor or oncogene in different cancer cells [16]. Other studies indicate that AMPK-mediated signaling elicits death in cancer cells via autophagy and/or apoptosis [17]. Therefore, it is intriguing to explore the role of the AMPK-mediated pathway in the cytotoxicity of CTXs. Prior studies have shown that human myeloid leukemia cells are highly susceptible to the cytotoxicity of CTXs [4,7,18]. Therefore, we investigated whether the AMPK-associated pathway is an important mediator in *N. atra* CTX3-induced death of human leukemia U937 cells.

## 2. Results

**Figure 1:**
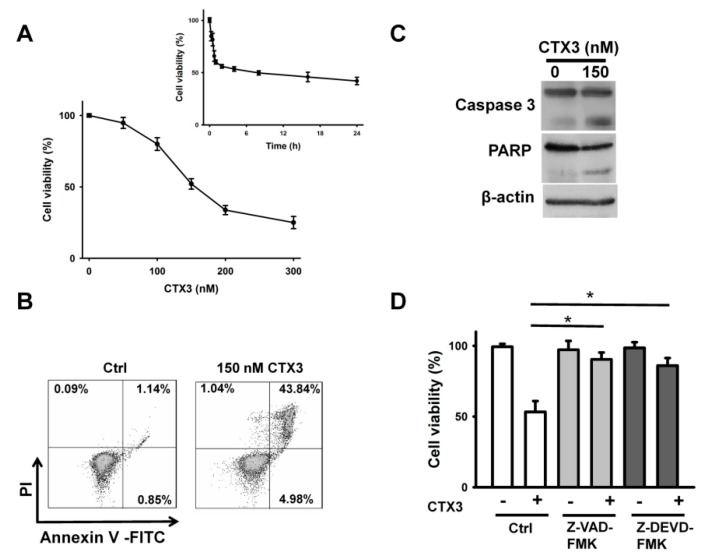
Cobra cardiotoxin (CTX)3 induced apoptotic death of U937 cells. (**A**) Effect of CTX3 on the viability of U937 cells. Cells were incubated with indicated CTX3 concentrations for 4 h. (Inset) U937 cells were treated with 150 nM CTX3 for indicated time periods. Cell viability was determined using methlythiazolyldiphenyl-tetrazolium bromide (MTT) assay. Results are expressed as the percentage of cell survival relative to the control. Each value is the mean ± SD of three independent experiments with triplicate measurements; (**B**) Flow cytometry analyses of annexin V-PI double staining CTX3-treated cells. U937 cells were incubated with 150 nM CTX3 for 4 h. On the flow cytometric scatter graphs, the left lower quadrant represents remaining live cells. The right lower quadrant represents the population of early apoptotic cells. The right upper quadrant represents the accumulation of late apoptotic cells; (**C**) Western blot analyses of procaspase-3 and poly(ADP-ribose) polymerase (PARP) degradation in CTX3-treated cells. U937 cells were incubated with 150 nM CTX3 for 4 h; (**D**) Viability of CTX3-treated cells was restored by pretreatment with caspase inhibitors. U937 cells were pretreated with 10 μM Z-VAD-FMK (pan-caspase inhibitor) or Z-DEVD-FMK (caspase-3 inhibitor) for 1 h, and then incubated with 150 nM CTX3 for 4 h. Each value is the mean ± SD of three independent experiments with triplicate measurements (* *p* < 0.05).

Treatment with CTX3 reduced the U937 survival of cells in a concentration- and time-dependent manner with an IC_50_ value of approximately 150 nM for a 4-h treatment (Figure 1A). Hence, we used this dose of CTX3 to study the mechanism of its cytotoxicity. CTX3 treatment increased the number of annexin V-FITC staining U937 cells (Figure 1B). In line with this, the CTX-treated cells showed degradation of procaspase-3 and PARP (Figure 1C), whereas the caspase inhibitors inhibited the cell death induced by CTX3 (Figure 1D). These results indicated that CTX3 induces apoptosis in U937 cells.

**Figure 2:**
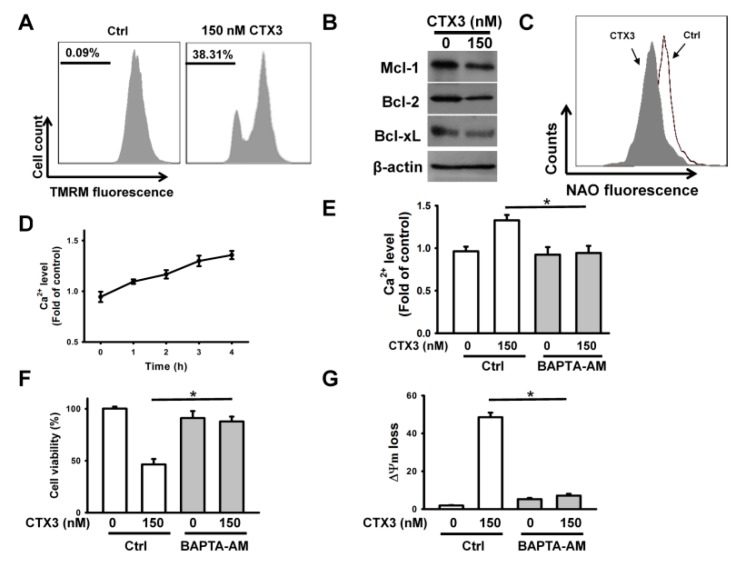
CTX3-induced increase in [Ca^2+^]i elicited mitochondrial depolarization. Without specific indication, U937 cells were treated with 150 nM CTX3 for 4 h. (**A**) CTX3 induced dissipation of ΔΨm. The loss of ΔΨm was analyzed by flow cytometry.; (**B**) Western blot analyses of Bcl-2, Bcl-xL, and Mcl-1 in CTX3-treated U937 cells.; (**C**) CTX3 caused a reduction in mitochondrial mass.; (**D**) CTX3 induced an increase in [Ca^2+^]i. U937 cells were incubated with 150 nM CTX3 for indicated time periods. Results were shown as fold-increase in fluorescence intensity compared with the control group. Each value is the mean ± SD of three independent experiments with triplicate measurements. (**E**) Effect of BAPTA-AM on CTX3-induced increase in [Ca^2+^]i in U937 cells. U937 cells were pretreated with 10 μM BAPTA-AM for 1 h, and then incubated with 150 nM CTX3 for 4 h (mean ± SD, * *p* < 0.05).; (**F**) Effect of BAPTA-AM on the viability of CTX3-treated cells (mean ± SD, * *p* < 0.05). (**G**) Effect of BAPTA-AM on CTX3-induced the loss of ΔΨm (mean ± SD, * *p* < 0.05).

To examine whether CTX3-induced apoptosis is related to mitochondrial dysfunction, the mitochondrial membrane potential (ΔΨm) of CTX3-treated cells was thus measured using TMRM fluorescence. CTX3 caused a marked loss of ΔΨm in U937 cells, as demonstrated by flow cytometry analysis (Figure 2A). Mcl-1, Bcl-2, or Bcl-xL suppression has been shown to induce mitochondrial permeability and ΔΨm loss [19]. Immunoblotting analyses showed that CTX3 induced downregultion of Mcl-1, Bcl-2, and Bcl-xL expression in U937 cells (Figure 2B). Moreover, we used 10-N-nonyl acridine orange (NAO), that binds to cardiolipin on the mitochondrial membrane, to measure the mitochondrial mass. Compared to the untreated control cells, the CTX3-treated cells showed a reduction in mitochondrial mass (Figure 2C). These results suggested that mitochondrial function is dysregulated in CTX3-treated U937 cells. As the cytotoxicity of CTXs is reported to be related to an increase in [Ca^2+^]i [12], we analyzed [Ca^2+^]i in U937 cells after CTX3 treatment. Compared to the untreated cells, the CTX3-treated cells showed a notable increase in [Ca^2+^]i (Figure 2D). Pretreatment with the intracellular calcium chelator, BAPTA-AM, inhibited the increases of [Ca^2+^]i and the death of U937 cells induced by CTX3 (Figure 2E,F). Moreover, BAPTA-AM alleviated the CTX3-induced dissipation of ΔΨm (Figure 2G). These results suggested a causal role of Ca^2+^ in CTX3-induced death of U937 cells.

**Figure 3:**
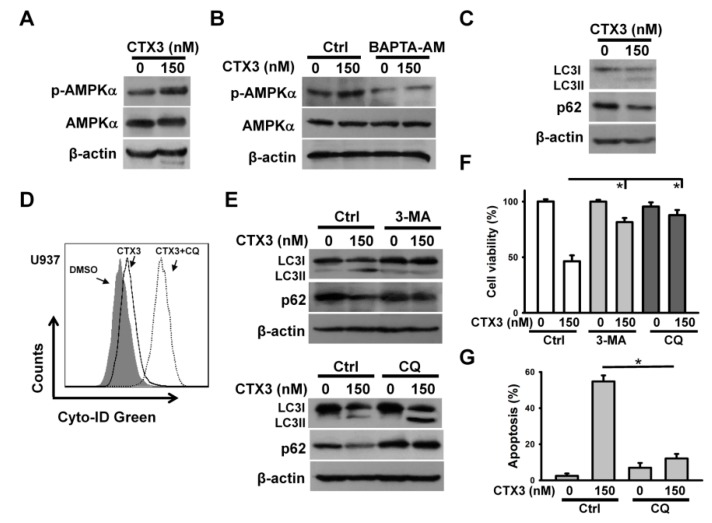
CTX3 induced autophagy of U937 cells. Without specific indication, U937 cells were treated with 150 nM CTX3 for 4 h. On the other hand, U937 cells were pre-treated with 10 μM BAPTA-AM, 10 μM 3-methyladenine (3-MA) or 10 μM chloroquine (CQ) for 1 h, and then incubated with 150 nM CTX3 for 4 h. (**A**) Western blot analyses of AMPKα and phospho-AMPKα in U937 cells; (**B**) Effect of BAPTA-AM on AMPKα phosphorylation; (**C**) Effect of CTX3 on LC3II and p62 expression; (**D**) Flow cytometry analysis of acidic vesicular organelles in cells treated with CTX3 or CQ plus CTX3. CTX3-treated cells were then stained with a Cyto-ID^TM^ autophagy detection kit according to manufacturer’s protocol; (**E**) Effect of 3-MA and CQ on CTX3-induced the formation of LC3II and p62 downregulation; (**F**) Effect of 3-MA and CQ on the viability of CTX3-treated cells. Cell viability was determined using MTT assay (mean ± SD, * *p* < 0.05). (**G**) Effect of CQ on CTX3-induced apoptosis of U937 cells. Apoptosis was assessed in triplicate by annexin V-PI double staining followed by flow cytometry, and percentage apoptosis is shown as percentage of annexin V-positive cells. Data represent mean ± SD (* *p* < 0.05).

Previous studies have revealed that AMPK has a role in suppressing the cytotoxicity of *N. pallida* CTX on C2C12 myoblast cells [12]. Moreover, AMPK can be activated by calcium signaling [17]. AMPKα phosphorylation at Thr172 represents AMPK activation. Thus, the AMPKα phosphorylation was examined. As shown in Figure 3A, CTX3 treatment increased the AMPKα phosphorylation. Pretreatment with BAPTA-AM inhibited the CTX3-induced AMPKα phosphorylation, suggesting that Ca^2+^ stimulated AMPK activation in the CTX3-treated cells. In addition to apoptosis (Figure 3B), AMPK-mediated signaling can elicit autophagy in cancer cells [17]. Thus, we analyzed the autophagy-related proteins, LC3 and p62. Figure 3C shows that the amount of LC3II was increased in the CTX3-treated U937 cells. CTX3 treatment reduced the p62 protein level. Flow cytometric analyses, using the Cyto-ID^TM^ autophagy detection kit, revealed an increase in acidic vesicular organelles in the CTX3-treated cells, compared to that in the untreated cells (Figure 3D). Further increase in the population of autophagic cells was observed when U937 cells were co-treated with CTX3 and chloroquine (CQ) (Figure 3D). The inhibition of autophagy by 3-methyladenine (3-MA) reduced the production of LC3II and restored p62 expression in the CTX-treated U937 cells (Figure 3E). CQ-induced inhibition of lysosomal degradation increased the expression of LC3II and p62 in the CTX3-treated U937 cells (Figure 3E). These results corroborated the CTX3-induced autophagy in U937 cells. Thus, 3-MA and CQ protected U937 cells from CTX3-induced cell death (Figure 3F). Pretreatment with CQ alleviated CTX3-induced apoptotic death (Figure 3G). These results indicated that CTX3-induced autophagy promoted the apoptotic death of U937 cells. 

**Figure 4:**
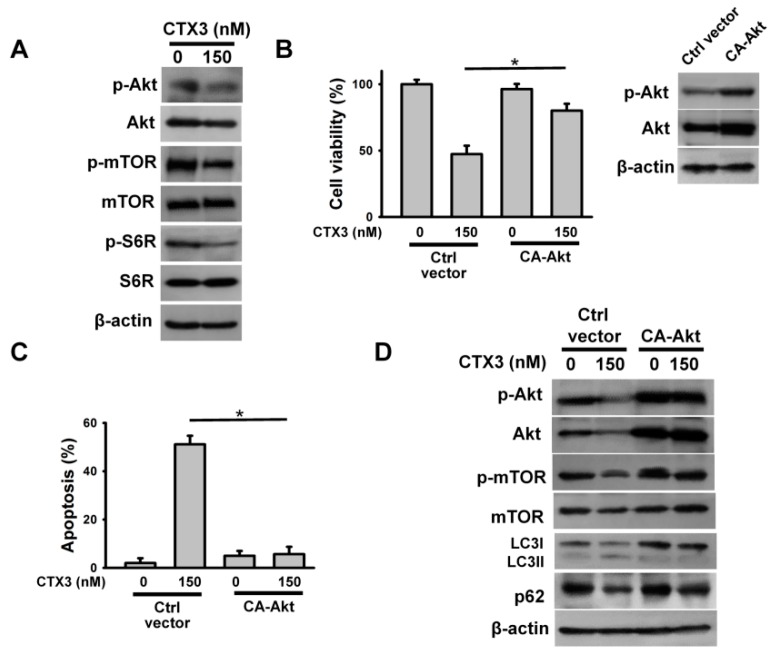
CTX3 induced phosphorylation of Akt, mTOR and S6R in U937 cells. Without specific indication, U937 cells were treated with 150 nM CTX3 for 4 h. (**A**) Effect of CTX3 on phosphorylation of Akt, mTOR, and S6R; (**B**) Effect of constitutively active Akt (CA-Akt) on the viability of CTX3-treated cells. U937 cells were transfected with empty expression vector or CA-Akt, respectively. After 24 h post-transfection, the transfected cells were treated with 150 nM CTX3 for 4 h. (Inset) Western blot analyses of Akt and phospho-Akt in CA-Akt-transfected cells; (**C**) Effect of CA-Akt on CTX3-induced apoptosis of U937 cells. Data represent mean ± SD (* *p* < 0.05). (**D**) Effect of CA-Akt on CTX3-induced the formation of LC3II and p62 downregulation.

Prior studies have shown that activated AMPK-induced autophagy is related to inhibition of the Akt/mTOR pathway [17]. Thus, we next analyzed the phosphorylation of Akt, mTOR, and S6R (mTORC1 substrate) in CTX3-treated U937 cells. Treatment with CTX3 reduced the phosphorylation level of Akt, mTOR, and S6R in U937 cells (Figure 4A). To verify that suppression of the Akt pathway is crucial for the cytotoxicity of CTX3, the effect of CTX3 on CA-Akt-overexpressing U937 cells was examined. As shown in Figure 4B, transfection of CA-Akt suppressed the cytotoxicity of CTX3 in U937 cells. Consistent with this, the overexpression of CA-Akt inhibited the CTX3-induced apoptosis of U937 cells (Figure 4C). Figure 4D shows that CTX3 was unable to inhibit mTOR phosphorylation in U937 cells expressing CA-Akt. Moreover, CA-Akt overexpression mitigated the effect of CTX3 on LC3II production and p62 downregulation in U937 cells. These results highlighted that CTX3-induced autophagy and apoptosis were mediated through suppression of the Akt/mTOR pathway.

**Figure 5:**
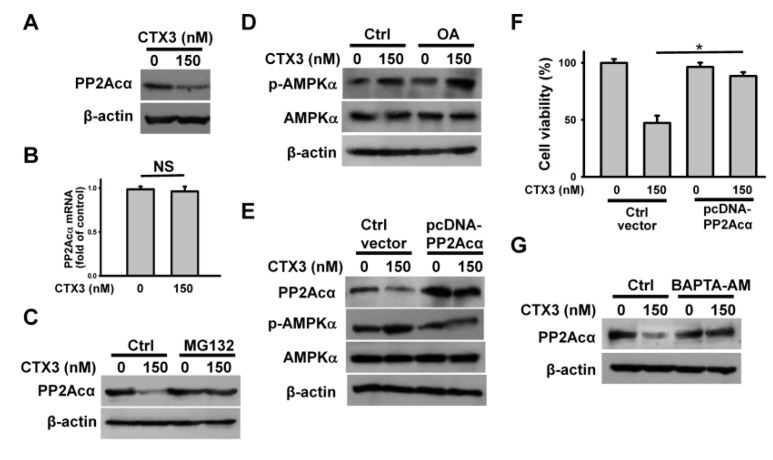
CTX3-induced PP2Acα degradation promoted AMPKα phosphorylation. Without specific indication, U937 cells were treated with 150 nM CTX3 for 4 h. On the other hand, U937 cells were pre-treated with 1 μM MG132, 10 nM okadaic acid (OA) or 10 μM BAPTA-AM for 1 h, and then incubated with 150 nM CTX3 for 4 h. (**A**) Western blot analyses of PP2Acα expression in CTX3-treated cells; (**B**) Quantitative analyses of PP2Acα mRNA level in CTX3-treated cells; (**C**) Effect of MG132 on PP2Acα expression in CTX3-treated cells; (**D**) Effect of OA on CTX3-induced AMPKα phosphorylation. (**E**) Effect of PP2Acα overexpression on AMPKα phosphorylation in CTX3-treated cells. U937 cells were transfected with empty expression vector or pcDNA-PP2Acα, respectively. After 24 h post-transfection, the transfected cells were treated with 150 nM CTX3 for 4 h. (**F**) Effect of PP2Acα overexpression on the viability of CTX3-treated cells (mean ± SD, * *p* < 0.05). (**G**) Effect of BAPTA-AM on PP2Acα expression in CTX3-treated cells.

Previous studies have shown that protein phosphatase 2A (PP2A) negatively regulate AMPK activation [20,21]. Moreover, downregulation of the PP2A catalytic subunit (PP2Ac) by microRNA-429 activates AMPK in osteoblastic cells [22]. We thus analyzed the expression of the PP2A catalytic subunit, PP2Acα, in the CTX3-treated cells. As shown in Figure 5A, CTX3 induced PP2Acα downregulation in U937 cells. However, quantitative RT-PCR analyses showed that the PP2Acα mRNA levels remained unchanged after CTX3 treatment (Figure 5B). Inhibition of proteasome activity by MG132 mitigated the CTX3-induced PP2Acα downregulation (Figure 5C), suggesting that CTX3 induced PP2Acα degradation. Co-treatment with okadaic acid and CTX3 showed a further increase in AMPKα phosphorylation in U937 cells compared to that with CTX3 treatment alone (Figure 5D). Conversely, overexpression of PP2Acα abolished CTX3-induced AMPKα phosphorylation (Figure 5E). These results indicate that the suppression of PP2A elicited AMPK activation in the CTX3-treated U937 cells. Moreover, overexpression of PP2Acα inhibited the cell death induced by CTX3 (Figure 5F), indicating the link between the PP2A-AMPK axis and CTX3 cytotoxicity. Figure 5G shows that BAPTA-AM pretreatment restored PP2Acα expression in the CTX3-treated cells. These results indicated the positioning of Ca^2+^ as a major regulator of CTX3-induced PP2Acα degradation.

**Figure 6:**
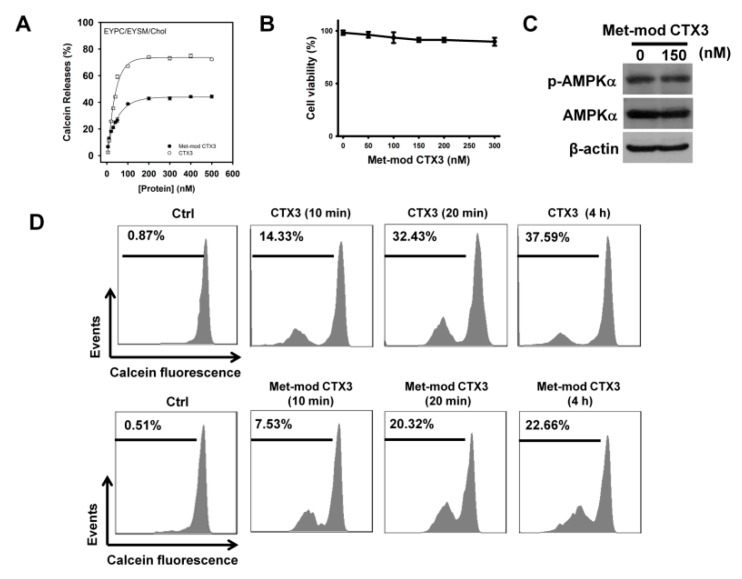
Effect of Met-modified CTX3 on AMPKα phosphorylation and membrane permeability of U937 cells. Without specific indication, U937 cells were treated with 150 nM CTX3 or Met-modified CTX3 for 4 h. (**A**) CTX3 and Met-modified CTX3 induced membrane permeability of EYPC/EYSM/cholesterol vesicles. Each value is the mean ± SD of three independent experiments with triplicate measurements; (**B**) Effect of Met-modified CTX3 on the viability of U937 cells. (**C**) Effect of Met-modified CTX3 on AMPKα phosphorylation in U937 cells; (**D**) CTX3 and Met-modified CTX3 induced the release of calcein from calcein-loaded U937 cells.

Prior studies suggest that the plasma membrane is a target of CTXs [9]. Compared to CTX3, Met-modified CTX3 showed a lower activity on the release of calcein from liposomes [23]. To evaluate whether CTX3 exerted its cytotoxicity through membrane damage, the activities of native and Met-modified CTX3 were analyzed comparatively. Met-modified CTX3 retained an approximate 50% activity of CTX3 on inducing the leakage of EYPC/EYSM/cholesterol vesicles (Figure 6A), whereas Met-modified CTX3 did not reduce the survival of U937 cells appreciably (Figure 6B). Treatment with Met-modified CTX3 did not induce AMPKα phosphorylation in U937 cells (Figure 6C). We further analyzed CTX3- and Met-modified CTX3-induced membrane permeability of calcein-loaded U937 cells. Treatment with CTX3 for 10 min notably increased the cell population with a reduced calcein fluorescent signal (Figure 6D). Exposure of calcein-loaded U937 cells to CTX3 for 20 min or up to 4 h resulted in calcein release to a similar extent (Figure 6D), showing that CTX3 did not further increase the membrane permeability of U937 cells after treatment for 20 min. Compared to CTX3, Met-modified CTX3 showed a lower activity on inducing the membrane permeability of the calcein-loaded U937 cells. However, a marked release of intracellular calcein was still noted after treatment of the calcein-loaded U937 cells with Met-modified CTX3 for 20 min. Considering that Met-modified CTX3 retained the capability to damage the membrane, these results suggested that the membrane-perturbing activity of CTX3 could not exclusively elucidate its cytotoxicity on U937 cells.

**Figure 7:**
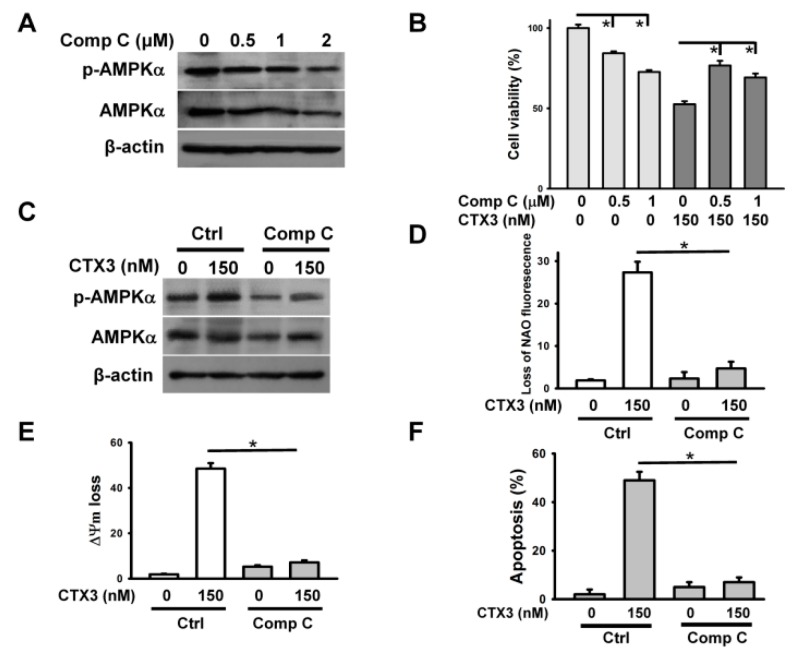
Effect of compound C on the cytotoxic effects of CTX3 on U937 cells. Without specific indication, U937 cells were pretreated with 0.5 μM compound C concentrations for 1 h, and then incubated with 150 nM CTX3 for 4 h. (**A**) Effect of compound C on AMPKα phosphorylation in U937 cells. U937 cells were pretreated with indicated compound C concentrations for 4 h. (**B**) Effect of compound C on the viability of CTX3-treated cells (mean ± SD, * *p* < 0.05). (**C**) Effect of compound C on AMPKα phosphorylation in CTX3-treated U937 cells.; (**D**) Effect of compound C on CTX3-induced reduction in mitochondrial mass. (**E**) Effect of compound C on CTX3-induced the loss of ΔΨm (mean ± SD, * *p* < 0.05). (**F**) Effect of compound C on CTX3-induced apoptosis of U937 cells (mean ± SD, * *p* < 0.05).

As our data showed that AMPKα phosphorylation is associated with the cytotoxicity of CTX3, we next analyzed the effect of compound C (AMPK inhibitor) on CTX3 cytotoxicity. Treatment with 0.5, 1, or 2 μM compound C reduced AMPKα phosphorylation and AMPKα expression in U937 cells (Figure 7A). U937 cells exposed to 0.5 or 1 μM compound C showed a reduction in viability by approximately 20% and 30%, respectively (Figure 7B). Thus, we only studied the effect of 0.5 and 1 μM compound C on CTX3 cytotoxicity. Combined treatment with 0.5 μM or 1 μM compound C and CTX3 protected the U937 cells from CTX3-induced cell death (Figure 7B). Co-treatment with 0.5 μM compound C inhibited CTX3-induced AMPKα phosphorylation (Figure 7C). Moreover, combination with 0.5 μM compound C mitigated the CTX3-induced the reduction in the mitochondrial mass, ΔΨm loss, and apoptosis (Figure 7D–F). These results highlighted that CTX3-induced AMPK activation is crucially involved in its cytotoxicity. 

## 3. Discussion

Our data reveal that CTX3 exerts its cytotoxicity on U937 cells via the Ca^2+^/PP2A/AMPK signaling pathway, and that the membrane-perturbing activity of CTX3 is not crucial for its capability to induce death in U937 cells. In agreement, previous studies have shown the distinct mechanism responsible for the cytotoxicity and membrane-damaging activity of CTX3 [24]. Although the internalization of CTXs into cells is suggested to be involved in their cytotoxicity [4,7,9,10,11], other studies suggest that *N. atra* CTX3 induces apoptosis of human CD8^+^ T lymphocytes via a membrane receptor-mediated signaling pathway [25]. Considering that Met-modified CTX3 retains its membrane-perturbing activity but does not elicit the AMPK-mediated cell death pathway, we deduced that CTX3-induced activation of death signaling in U937 cells is not exclusively dependent on the process of toxin internalization.

Some studies have proposed that internalization of *N. atra* CTX3 and *N. mossambica mossambica* CTXs elicits mitochondrial fragmentation in H9C2 myoblast cells, primary cortical neurons, or SH-SY5Y neuroblastoma cells [10,11]. Other studies have reported that an increase in lysosomal membrane permeability and the release of lysosomal cathepsin B elucidates the cytotoxic effect of *N. atra* CTX1 on breast cancer MCF-7 and leukemia K562 cells [7]. Feofanov et al. [4] suggested that the capability of internalized *N. oxiana*, *N, kaouthia*, and *N. haje* CTXs to disrupt lysosome determines their cytotoxicity in human lung cancer A549 cells and leukemia HL60 cells. In contrast, Wang et al. [26] found that the internalized *N. atra* CTX3 induces apoptosis in fetal rat primary neurons, whereas it induces necrosis in fetal rat primary cardiomyocytes and astrocytes. Other studies showed that *N. nigricollis* CTX, toxin γ, notably induces mitochondria-mediated death pathway in U937 cells, whereas it shows lower cytotoxicity against human peripheral blood mononuclear cells [27]. These results suggested that (1) CTX isotoxins do not share the same intracellular targets, and/or (2) the cytotoxic targets of CTXs are cellular context- and cell-type dependent. Thus, we deduce that the Ca^2+^/PP2A/AMPK axis identified in the present study cannot fully elucidate the mechanisms responsible for the cytotoxicity of CTX isotoxins in different types of cancer cells or normal cells. Nevertheless, previous studies have demonstrated that AMPK activators induce mitochondrial fragmentation [14,28]. Sekar et al. [29] found that the AMPK activator, P2X7, exerts its cytotoxicity in microglia cells via induction of lysosomal instability and cathepsin B release. Altogether, the involvement of AMPK in CTX-induced mitochondrial fragmentation and lysosomal disruption is worth investigating further.

Previous studies have shown that metformin (AMPK activator) repressed the cytotoxicity of *N. pallida* CTX on C2C12 myoblast cells [12]. On the contrary, our data reveal that CTX3-induced AMPK activation elicits autophagy-dependent apoptosis in U937 cells. Supplementary Figure S1A shows that treatment with 1, 5, or 10 mM metformin all notably increase AMPKα phosphorylation. Co-treatment with 1 mM metformin further increased AMPKα phosphorylation in CTX3-treated cells, whereas combined treatment with metformin enhanced the CTX3-induced death of U937 cells (Supplementary Figure S1B,C). Similarly, treatment with 1, 5, or 10 μM A-769662 (AMPK activator) increased AMPKα phosphorylation ( Supplementary Figure S1D), and co-treatment with 5 μM A-769662 further increased the AMPKα activation in CTX3-treated cells (Supplementary Figure S1E). Consistently, co-treatment with A-769662 enhanced the cytotoxicity of CTX3 in U937 cells (Supplementary Figure S1F). Evidently, the AMPK activator did not inhibit the CTX3-induced death of U937 cells. Thus, the hypothesis that CTX isotoxins induce cellular context- or cell-type dependent death signaling remains a subject of future investigation.

Prior studies have shown that α4 plays a crucial role in stabilizing PP2Ac and protects PP2Ac from poly-ubiquitination and degradation by MID1 [30,31,32]. We analyzed α4 and MID1 expression in CTX3-treated U937 cells, and found that CTX3 reduced α4 expression but not MID1 expression (Supplementary Figure S2A). Pretreatment with BAPTA-AM restored PP2Acα (Figure 5G) and α4 expression in CTX3-treated cells (Supplementary Figure S2B). These results suggest that the CTX3-induced elevation in [Ca^2+^]i distorts α4-mediated PP2Acα stabilization. 

## 4. Conclusions

In conclusion, we identified that the cytotoxicity of *N. atra* CTX3 on U937 cells is related to the Ca^2+^/PP2A/AMPK axis-elicited autophagy and apoptosis. Additionally, our data suggest a dissociation of CTX3-induced death signaling from its effect on cell membrane damage.

## 5. Materials and Methods

### 5.1. Chemicals and Reagents

Purification of *N. atra* CTX3 and modification of Met residues in CTX3 were performed according to the procedure described previously [33]. Calcein, egg yolk phosphatidylcholine (EYPC), egg yolk sphingomyelin (EYSM), cholesterol, MTT, MG132, chloroquine, 3-methyladenine, metformin, A-769662, and anti-β-actin antibody were purchased from Sigma-Aldrich Inc. (St. Louis, MO, USA); and calcein-AM, Fluo-4 AM, BAPTA-AM, and tetramethylrhodamine methylester (TMRM) were from Molecular Probes (Eugene, OR, USA). Cyto-ID^TM^ autophagy detection kit was the product of Enzo Life Sciences Inc. (Farmingdale, NY, USA). Compound C was obtained from Cayman chemical Co (Ann Arbor, MI, USA). Annexin V-FITC/propidium iodide (PI) flow cytometry assay kit and 10-n-nonyl-acridinium-orange-chloride (NAO) were purchased from Invitrogen (Carlsbad, CA, USA); and okadaic acid, Z-DEVD-FMK and Z-VAD-FMK were from Calbiochem (San Diego, CA, USA). Antibodies separately against BCL2, MCL1, and PP2Ac were obtained from Santa Cruz Biotechnology (Santa Cruz, CA, USA); antibodies separately against caspase-3, α4, MID1, PARP, BCL2L1, AMPKα, p-AMPKα, LC3B, p62, mTOR, p-mTOR, S6R, p-S6R, Akt, and p-Akt were from Cell Signaling Technology (Beverly, MA, USA). HRP-conjugated rabbit anti-mouse IgG and goat anti-rabbit IgG were the products of Pierce (Rockford, IL, USA). Cell culture supplements were from GIBCO/Life Technologies Inc. (Carlsbad, CA, USA). 

### 5.2. Cell Culture

Human acute myeloid leukemia U937 cells were cultured in RPMI-1640 medium containing 10% FCS (fetal calf serum), 1% sodium pyruvate, 2 mM L-glutamine, penicillin (100 units/mL), and streptomycin (100 μg/mL) incubating at 37 °C in an humidified incubator with 5% CO_2_ atmosphere. 

### 5.3. Measurement of Mitochondrial Depolarization

CTX3-treated cells were incubated with 2 nM TMRM for 20 min following by washing with PBS. Fluorescence intensity of TMRM was analyzed using flow cytometry. The cell population with the loss of ΔΨm showed a reduction in TMRM fluorescence.

### 5.4. Measurement of Intracellular Ca^2+^ Concentration ([Ca^2+^]i)

After incubation of CTX3-treated cells with 5 μL of 2 μM Fluo-4 AM for 1 h, the fluorescence of [Ca^2+^]i was detected using Beckman Coulter ParadigmTM Detection Platform.

### 5.5. Measurement of Mitochondrial Mass

CTX3-treated cells were incubated with 10 nM NAO for 20 min at 37 °C. The NAO fluorescence was measured using a flow cytometer, and mitochondrial mass was evaluated using NAO fluorescent intensity.

### 5.6. Quantitative RT-PCR

Quantitative RT-PCR was performed to detect the levels of PP2Acα mRNA using GoTag qPCR Master mix (Promega, Madison, WI). Primer sequences are listed in following: PP2Acα, 5’-TCGTTGTGGTAACCAAGCTG-3’ (F), 5′-AACATGTGGCTCGCCTCTAC-3’ (R); GAPDH, 5’-GAAATCCCATCACCATCTTCCAGG-3’ (F), 5’-GAGCCCCAGCCTTCTCCATG-3’ (R). 

### 5.7. Transfection of DNA

The pcDNA-PP2Acα and constitutively activated, myristoylated AKT (CA-Akt) plasmids were described in our previous studies [34,35]. Transfection of U937 cells with the plasmids was conducted using 4D-Nucleofector (Lonza, Cologne AG, Germany).

### 5.8. Membrane Permeability Assay

EYPC/EYSM/cholesterol (mol/mol/mol, 49:21:30) vesicles encapsulating calcein at the self-quenching concentration (50 mM) was prepared according to previous report [22]. The lipid vesicles dispersed in 10 mM Tris-HCl-100 mM NaCl (pH 7.5) were used for analyzing membrane-damaging activity of CTX3. CTX3-induced the release of calcein was expressed as percentage of totally dequenched calcein fluorescence after the addition of 0.2% Triton X-100. The calcein fluorescence was measured using excitation and emission wavelength at 490 nm and 520 nm, respectively.

### 5.9. Leakage of Calcein-Loaded Cells

U937 cells were incubated with 5 μM calcein-AM for 1 h at 37 °C following by washing with 10 mM Tris-HCl-100 mM NaCl (pH 7.5). The cells were further incubated at 37 °C for 20 min for the conversion of non-fluorescent calcein-AM to fluorescent calcein inside the cells. After treatment with CTX3 or Met-modified CTX3, the fluorescent signal of calcein-loaded cells was analyzed using flow cytometer.

### 5.10. Other Tests

Cell viability analysis using MTT assay, flow cytometric analysis of apoptosis using annexin V-FITC/PI kit, qualification of acidic vesicular organelles using Cyto-ID^TM^ autophagy detection kit, and western blot analyses were performed according to the procedure previously described [36]. 

### 5.11. Statistical Analysis

Data were analyzed using GraphPad Prism (GraphPad software, version 5.01, San Diego, CA, USA). All data are expressed as mean ± SD. Differences among the means were assessed using one-way analysis of variance (ANOVA) followed by Tukey’s multiple comparison test. Difference considered significant when *p* < 0.05. 

## Supplementary Materials

The following are available online at https://www.mdpi.com/2072-6651/11/9/527/s1, Figure S1: Effect of metformin and A-769662 on CTX3-induced cell death and AMPKα phosphorylation. Without specific indication, U937 cells were treated with 150 nM CTX3 for 4 h. (A) Effect of metformin on AMPKα phosphorylation in U937 cells. U937 cells were incubated with indicated metformin concentrations for 4 h; (B) Metformin enhanced CTX3-induced AMPKα phosphorylation. U937 cells were co-treated with 1 mM metformin and 150 nM CTX3 for 4 h; (C) Metformin enhanced CTX3-induced cell death. U937 cells were co-treated with indicated metformin and CTX3 concentrations for 4 h (mean ± SD, * *p* < 0.05); (D) Effect of A-769662 on AMPKα phosphorylation in U937 cells. U937 cells were incubated with indicated A-769662 concentrations for 4 h; (E) A-769662 enhanced CTX3-induced AMPKα phosphorylation. U937 cells were co-incubated with 5 μM A-769662 and 150 nM CTX3 for 4 h; (F) A-769662 enhanced CTX3-induced cell death. U937 cells were incubated with indicated A-769662 and CTX3 concentrations for 4 h; Figure S2: Effect of CTX3 on α4 and MID1 expression in CTX3-treated cells. Without specific indication, U937 cells were treated with 150 nM CTX3 for 4 h. On the other hand, U937 cells were pre-treated with 10 μM BAPTA-AM for 1 h, and then incubated with 150 nM CTX3 for 4 h. (A) Western blot analyses of α4 and MID1 expression in CTX3-treated cells; (B) Effect of BAPTA-AM on α4 expression in CTX3-treated cells.
