# Supplementary material for: Naja atra Cardiotoxin 3 Elicits Autophagy and Apoptosis in U937 Human Leukemia Cells through the Ca2+/PP2A/AMPK Axis
Source: Toxins (Basel). 2019 Sep 12;11(9):527. doi: 10.3390/toxins11090527 (PMC6784133; doi:10.3390/toxins11090527)
Supplement: Supplementary file 1 [file toxins-11-00527-s001.pdf]

Supplementary Materials: *Naja atra* Cardiotoxin 3 Elicits Autophagy and Apoptosis in U937 Human Leukemia Cells through the Ca^{2+}/PP2A/AMPK Axis

Jing-Ting Chiou, Yi-Jun Shi, Liang-Jun Wang, Chia-Hui Huang, Yuan-Chin Lee and Long-Sen Chang

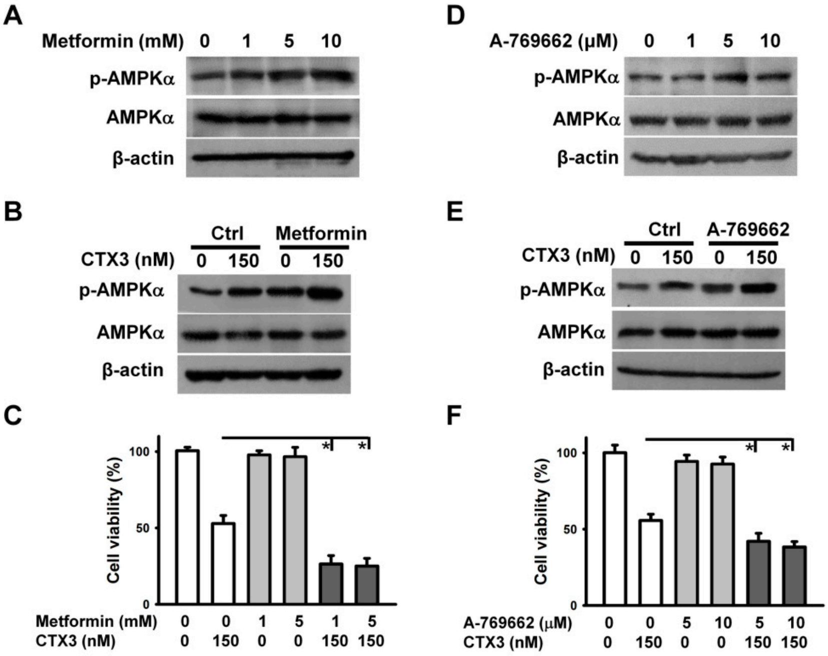

Figure S1. Effect of metformin and A-769662 on CTX3-induced cell death and AMPKα phosphorylation. Without specific indication, U937 cells were treated with 150 nM CTX3 for 4 h. (**A**) Effect of metformin on AMPKα phosphorylation in U937 cells. U937 cells were incubated with indicated metformin concentrations for 4 h; (**B**) Metformin enhanced CTX3-induced AMPKα phosphorylation. U937 cells were co-treated with 1 mM metformin and 150 nM CTX3 for 4 h; (**C**) Metformin enhanced CTX3-induced cell death. U937 cells were co-treated with indicated metformin and CTX3 concentrations for 4 h (mean ± SD, * $p < 0.05$); (**D**) Effect of A-769662 on AMPKα phosphorylation in U937 cells. U937 cells were incubated with indicated A-769662 concentrations for 4 h; (**E**) A-769662 enhanced CTX3-induced AMPKα phosphorylation. U937 cells were co-incubated with 5 μM A-769662 and 150 nM CTX3 for 4 h; (**F**) A-769662 enhanced CTX3-induced cell death. U937 cells were incubated with indicated A-769662 and CTX3 concentrations for 4 h.

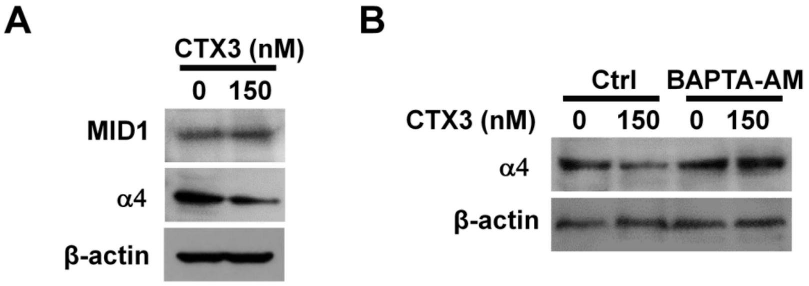

Figure S2. Effect of CTX3 on α4 and MID1 expression in CTX3-treated cells. Without specific indication, U937 cells were treated with 150 nM CTX3 for 4 h. On the other hand, U937 cells were pre-treated with 10 μM BAPTA-AM for 1 h, and then incubated with 150 nM CTX3 for 4 h. (**A**) Western blot analyses of α4 and MID1 expression in CTX3-treated cells; (**B**) Effect of BAPTA-AM on α4 expression in CTX3-treated cells.
